# Vaccines for the prevention of seasonal influenza in patients with diabetes: systematic review and meta-analysis

**DOI:** 10.1186/s12916-015-0295-6

**Published:** 2015-03-17

**Authors:** Cornelius Remschmidt, Ole Wichmann, Thomas Harder

**Affiliations:** Robert Koch Institute, Immunization Unit, Seestrasse 10, Berlin, 13353 Germany

**Keywords:** Diabetes, Effectiveness, Influenza, Meta-analysis, Systematic review, Vaccine

## Abstract

**Background:**

Patients with diabetes are at increased risk of severe influenza disease; influenza vaccination for these patients is therefore recommended by the World Health Organization and several National Immunization Technical Advisory Groups. However, no systematic review has evaluated the effects of influenza vaccines for patients with diabetes.

**Methods:**

We conducted a systematic review and meta-analysis by searching Medline, Embase, Cochrane Central Register of Controlled Trials, and ClinicalTrials.gov from inception until November 2014. We included all types of studies reporting on the efficacy, effectiveness, and/or safety of influenza vaccination in patients with type 1 and type 2 diabetes of all ages. We used the Newcastle-Ottawa scale to assess risk of bias in observational studies. Residual confounding was addressed by comparing estimates of vaccine effectiveness (VE) during influenza seasons to those obtained during off-seasons. Quality of the evidence for each outcome was assessed using the GRADE methodology.

**Results:**

Following review of 1,444 articles, 11 observational studies with a total of 170,924 participants were included. In diabetic patients of working-age (18–64 years), influenza vaccination prevented all-cause hospitalization with a pooled VE of 58% (95% CI, 6–81%) and hospitalization due to influenza or pneumonia (VE 43%; 95% CI, 28–54%), whereas no effects on all-cause mortality and influenza-like illness (ILI) were observed. In the elderly (65+), influenza vaccination prevented all-cause mortality (VE 38%; 95% CI, 32–43%), all-cause hospitalization (VE 23%; 95% CI, 1–40%), hospitalization due to influenza or pneumonia (VE 45%; 95% CI, 34–53%), and ILI (VE 13%; 95% CI, 10–16%). However, significant off-season estimates for several outcomes indicated residual confounding, particularly in elderly patients. Quality of the evidence was low to very low for all outcomes. Laboratory-confirmed influenza infections were not reported.

**Conclusions:**

Due to strong residual confounding in most of the identified studies, the available evidence is insufficient to determine the magnitude of benefit that diabetic people derive from seasonal influenza vaccination. Adequately powered randomized controlled trials or quasi-experimental studies using laboratory-confirmed influenza-specific outcomes are urgently needed.

**Electronic supplementary material:**

The online version of this article (doi:10.1186/s12916-015-0295-6) contains supplementary material, which is available to authorized users.

## Background

Worldwide, more than 347 million people live with diabetes [1]. Since patients with diabetes are at increased risk of medical complications attributable to influenza infections [2], annual vaccination against influenza is recommended for these patients by the World Health Organization (WHO) [3] and several National Immunization Technical Advisory Groups (NITAGs) [4-7]. However, the underlying pathology of greater susceptibility to influenza and its complications is not well understood. An impaired immune response has been hypothesized to be responsible for an increased risk of infection [8-10] as well as infection-related complications in patients with diabetes [11,12]. If this hypothesis holds true, the immune response to influenza vaccines might be impaired in diabetic patients as well. In fact, while some studies found a reduced immune response to vaccination in patients with diabetes [13,14], others did not detect a difference in the humoral response between diabetic patients and healthy controls [15-18]. However, the value of these studies is limited not only because of their small size and contradicting findings, but also because influenza antibody titers do not perfectly correlate with clinical protection [19]. Therefore, evidence from post-marketing studies with clinical outcomes is highly desirable.

Knowledge of the benefits and harms is important to inform decision-making for vaccination and crucial for public health authorities when defining vaccination target groups. As such, we performed a systematic review and meta-analysis on influenza vaccine efficacy/effectiveness (VE) and safety in diabetic patients of all ages. We draw particular attention on the analysis of bias and confounding and used the methodology developed by the Grading of Recommendations Assessment and Evaluation (GRADE) working group to assess the quality of evidence for each outcome.

## Methods

### Eligibility criteria

To be eligible for this systematic review, a study had to be an original report on the efficacy, effectiveness, and/or safety of vaccines against seasonal influenza in individuals with diabetes mellitus. Patients with type 1 and type 2 diabetes of all ages were included. The control group had to be either unvaccinated or must have received placebo. All reported clinical or laboratory-confirmed outcomes and all types of local and systemic adverse events were considered. No restrictions were made regarding study type, publication language, and publication status.

### Search strategy and data extraction

The systematic review was performed according to the Preferred Reporting Items for Systematic Reviews and Meta-analyses (PRISMA) statement [20]. The study protocol of this review is available in Additional file 1. The literature search was independently developed by two reviewers (CR and TH) and discussed with a librarian of the Robert Koch Institute. Two reviewers (CR and TH) independently searched MEDLINE, EMBASE, and Cochrane Central Register of Controlled Trials (date of last search: November 25, 2014) and the screening process was not different for citations versus full-text articles. For complete search strategy, see Additional file 2. In addition, ClinicalTrials.gov and reference lists of all identified studies were reviewed for additional studies.

From each eligible original study, two independent reviewers (CR and TH) extracted study characteristics and assessed methodological quality, using standardized forms. Extraction forms were pilot tested with the first identified study and disease-specific data (e.g., diagnosis of type of diabetes; type of therapy) were added. For one study [21], the corresponding author was contacted to resolve discrepancies in published data. For a study, which was published as a congress abstract [22], we contacted the authors to obtain further details. In case of disagreements regarding screening, data extraction, and quality assessment a final decision was made by consensus or resolved by a third reviewer (OW). The following information was extracted: country, year, study design, age at vaccination, sex, identification of diabetic patients, conflict of interest declared by study authors, vaccine used (name, manufacturer), number of vaccinated and unvaccinated participants, proportion of participants lost to follow up, relative risks, odds ratios, hazard ratios, risk difference for defined outcomes, confounder-adjusted estimates, confounders considered, and control period (off-season) estimates.

### Assessment of bias

The Cochrane Risk of Bias tool was used to assess risk of bias in randomized controlled trials (RCTs) and the Newcastle-Ottawa Scale was used to assess the risk of bias in quasi-experimental studies and in observational studies. Following the suggestions made by the Cochrane Collaboration [23], we assessed risk of bias separately for each outcome and expressed the results as a considered judgment, using the categories ‘high risk of bias’, ‘low risk of bias’, and ‘unclear risk of bias’. In addition, we assessed the risk of healthy vaccinee bias and confounding by indication in the included studies. Details of the methodology are described in Additional file 3.

### Assessment of quality of the body of evidence

We used the GRADE methodology to assess the quality of the respective body of evidence for each outcome [24,25]. According to GRADE, outcomes of an intervention are categorized into four levels of evidence quality: +very low, ++ low, +++ moderate, and ++++ high. In GRADE, bodies of evidence from RCTs start as high quality evidence, whereas those from observational studies start as low quality evidence. Defined criteria are applied to either decrease or increase quality of evidence rating. Details on the GRADE methodology can be found elsewhere [25-27]. Applying the principle of assessing the best available evidence, we assessed the results of the confounder-adjusted analyses with GRADE.

### Statistical analysis

Abstracted data were aggregated in tables, together with risk of bias assessments. Risk ratios, odds ratios, risk differences and corresponding 95% confidence intervals (95% CIs) were either calculated or extracted directly from the publications. VE was calculated as 1–(risk ratio comparing vaccine and control recipients) × 100.

All analyses were performed separately according to study design and age group. Where data from more than one study on a given outcome were available, we performed meta-analysis, using a random-effects model to account for heterogeneity. I^2^ was used to quantify the extent of heterogeneity. In addition to pooling crude estimates, we pooled confounder-adjusted estimates if they were adjusted at least for age, sex, and comorbidities. To evaluate the presence of residual confounding, we contrasted estimates of VE measured during the influenza season to estimates measured during ‘control periods’ outside the influenza season in the same studies. Using the confounder-adjusted estimate, residual confounding was defined to be present if vaccination had a statistically significant effect on a given outcome in the absence of influenza virus circulation (‘off-season’). Formal testing for publication bias was not done since study numbers for each outcome were too small. Calculations were done using STATA 12 (StataCorp LP, Texas, USA) and Review Manager (RevMan 5.2, Cochrane Collaboration). The results of the GRADE evidence rating were recorded in GRADE evidence profiles using the GRADEpro software [28].

## Results

### Selection of studies

**Figure 1:**
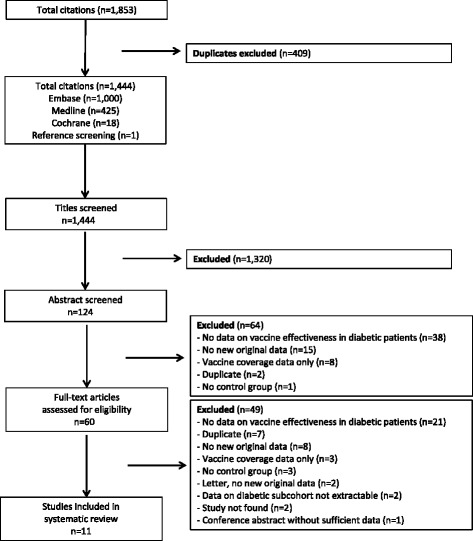
**Flow chart for the systematic literature search and study selection related to efficacy, effectiveness, and safety of influenza vaccines in diabetic patients.**

After removal of duplicates, we identified 1,444 records in electronic databases (Figure 1) and finally included a total of 11 original studies comprising data of 170,924 diabetic patients. Of these, 10 were identified via databases and one study [29] was identified from a reference list. In addition, we identified one conference abstract [22] which, however, did not provided sufficient data to be extracted. We did not identify further unpublished data. All studies were published in English. The main reasons for exclusion were that no data on vaccine effectiveness or vaccine safety in diabetic patients were reported, that studies did not provide original data (e.g., narrative review or guidelines), or that only vaccine coverage data on diabetic patients were provided (details on excluded studies are reported in Additional file 4). No additional studies reporting on influenza VE or safety specifically in subgroups of diabetic patients were identified in the Cochrane review on influenza vaccination in the elderly [30].

### Study characteristics

We included six cohort [21,29,31-34] and five case–control [35-39] studies. No RCTs or other experimental or quasi-experimental studies were identified. Six studies [21,29,32,34,37,39] included patients aged 65 or older and two case–control studies [35,38] provided stratified analyses on both elderly and adults of working-age (18 to 64 years). None of the studies presented data on children or adolescents. One article [31] did not report the age of included patients (Table 1).

**Table 1:** **Characteristics of included studies on influenza vaccine effectiveness in diabetic patients**

**Author**	**Study design, year**	**Country**	**Age in years (mean or range)**	**Percentage male**	**Identification of diabetic patients**	**Predominantly circulating influenza (sub)strains**	**Study size (n)**
**Cohort studies**							
Hak [29]	Prospective, 1996/1997 and 1997/1998	US	≥65, but not reported for diabetic sub-cohort	Not reported for diabetic sub-cohort	ICD-9 codes, database of managed care organizations	A(H3N2) in both seasons	14,915 in 96/97
							21,991 in 97/98
Heymann [21]	Retrospective, 2000/2001	Israel	Vacc., 72.8 ± 0.6	Vacc., 51.8	ICD-9 codes, diabetes register of healthcare service	Not reported	16,383
			Non-vacc., 73.1 ± 0.5	Non-vacc., 42.1			
Isotani [31]	Retrospective, 1999/2000	Japan	Not reported	Not reported	Outpatient clinic, unclear	Influenza A	450
Rodriguez-Blanco [32]	Retrospective, 2002–2005	Spain	Vacc., 75.2 ± 6.5	Vacc., 39.8	ICD-9 codes, clinical records	A(H3N2)	2,650
			Non-vacc., 73.1 ± 6.9	Non-vacc., 42.2			
Selvais [33]	Prospective, 1995/96	Belgium	56.3 ± 15.9 years	55	Diabetes clinic, unclear	Not reported	432
Schade [34]	Retrospective, 1995–1997	US	65 to 114 years	Not reported	ICD-9 codes, hospital discharge	Not reported	26,443 in 96/97
							23,839 in 97/98
**Case–control studies**							
Colquhoun [36]	not matched, 1989 and 1993	UK	≤19–85 years	Cases, n = 59	ICD-9 codes, diabetes register	1989, A(H3N2)	114 (37 cases, 77 controls)
				Controls, n = 55		1993, A(H3N2) and B	
Gasparini [37]	Matched, 2010/11	Italy	Not reported for diabetic sub-cohort	Not reported for diabetic sub-cohort	ICD-9 codes, hospital discharge	A(H1N1)pdm, and B	78 (46 cases, 32 controls)
Lau^2^ [35]	Nested, 2000–2008	Canada	Working-age, 53	Working age, 52 elderly, 47	ICD-9/10 codes, administrative database	Not reported	91,605
			Elderly, 74^1^				
Looijmans [38]	Nested, 1999/2000	Netherlands	Cases, 68.1 ± 13.7	Cases, 51.6 controls, 38.3	ICPC codes, general practitioners	A(H3N2)	1,753 (192 cases, 1,561 controls)
			Controls, 69.8 ± 12.6				
Wang [39]	Nested, 2001–2009	Taiwan	Vacc., 73.1 ± 5.9	Vacc, 50.0	ICD-9 codes, NHRI-database	Not reported	9,025 (4,571 vacc., 4,454 non-vacc.)
			Non-vacc., 73.2 ± 6.8	Non-vacc., 49.5			
**Total population**							**170,924** ^3^

^1^Median; ^2^Published as cohort study, however, due to matching of controls, this study was considered as case–control study here; ^3^Since overlapping of the study population reported by Hak et al. and Schade et al. for two influenza seasons cannot be ruled out for calculation of the total population for each study numbers of only one season were used.

ICD, International classification of diseases codes; ICPC, International Classification of Primary Care Codes; NHRI, Claims data from the National Health Research Institutes Database.

Nine of 11 studies used either International Classification of Diseases Codes (ICD-9/10) or International Classification of Primary Care Codes from clinical records or administrative databases to identify diabetic patients and subsequent influenza disease. Only one study provided data on the type of diabetes, glycemic control, and number of years that patients lived with diabetes [36]. One study reported on three different vaccine types that were used by included patients [37]. However, a stratified analysis (e.g., VE of adjuvanted vs. non-adjuvanted vaccines) was not possible, since diabetic patients were not stratified accordingly.

Five studies gave information on potential conflicts of interest [32,35,37-39]. Of those, in one study, a co-author received consulting fees and had served on an advisory board of a pharmaceutical company [38].

### Reported outcomes

Overall, six different clinical outcomes were reported. Outcomes that were addressed by at least one of the studies were all-cause mortality, all-cause hospitalization, hospitalization due to influenza or pneumonia (based on hospital discharge diagnosis codes), influenza-like illness (ILI), admission to an intensive care unit (ICU), and respiratory failure (Additional file 5). Since it was unclear whether the outcomes ICU-admissions and respiratory failures reported in one study [39] were a subgroup analysis of hospitalized cases, these outcomes were excluded from the final analysis. Two studies additionally provided data on the compound outcome ‘hospitalization or death’ [29,38]. None of the studies reported data on vaccine safety and none of the studies gave data on laboratory-confirmed influenza infections. Four of 11 studies did not report adjusted VE estimates [21,31,33,37] and one study did not provide crude point estimates [35]. Three studies measured ‘pseudo-effectiveness’ of influenza-related outcomes off-season [21,32,35].

### Risk of bias assessment in individual studies

Outcome-specific risk of bias assessment is presented in Additional file 6. In six cohort studies, eight estimates were reported. In two cohort studies, risk of bias was low regarding all-cause mortality and the compound outcome hospitalization or death, respectively. The remaining estimates, which were reported in cohort studies, had a high risk of bias.

In the five case–control studies, 18 estimates for different outcomes were reported. Six estimates presented by four studies indicated high risk of bias, whereas the remaining 12 estimates were at low risk (Additional file 6). Notably, in one study [35] risk of bias differed within the study among different outcomes and age groups: in adults of working-age, risk of bias was high for all-cause hospitalization but low for hospitalization due to influenza or pneumonia and for ILI, whereas, among the elderly, risk of bias was high for all these outcomes.

In 7 of the 11 included studies confounding by indication was likely (Additional file 3): baseline characteristics showed that vaccinated participants had more comorbidities than unvaccinated participants. Healthy vaccinee bias, however, was likely to be present in a subgroup of patients in one study only; Lau et al. [35] showed that, among the elderly, vaccinated patients had fewer comorbidities than unvaccinated participants.

### Vaccine effectiveness in adults aged 18 to 64 years

Overall, three case–control and one cohort study presented data on working-age adults. Crude and adjusted odds ratios for single studies for all outcomes are shown in Additional file 5. In the pooled analysis (Table 2), adjusted point estimates correspond to VE of 58% (95% CI, 6–81%, I^2^ = 77%, n = 3) against all-cause hospitalization. Point estimates regarding hospitalization due to influenza or pneumonia were reported by only one study and corresponded to a VE of 43% (95% CI, 28–54%, n = 1). Regarding all-cause mortality and ILI, no statistically significant protective effects were observed; however, adjusted estimates for each of the latter three outcomes were reported by only one study. Off-season VE estimates against all-cause hospitalization calculated in one study were lower than estimates during influenza seasons, but still showed a protective effect (VE, 27% (95% CI, 17–35%, n = 1)), thereby indicating residual confounding. Off-season VE estimates regarding other outcomes were not statistically significant.

**Table 2:** **Pooled crude and adjusted odd ratios of influenza vaccine effectiveness (per outcome) during influenza-season and off-season in vaccinated vs. unvaccinated diabetic patients**

**Age-group (years)**	**Outcome/Design**		**No of studies included**	**Crude OR (95% CI)**	**I** ^**2**^	**No of studies included**	**Adjusted OR (95% CI)**	**I** ^**2**^	**No of studies included**	**Off-season adjusted OR (95% CI)**	**I** ^**2**^
**0–17**	**No studies**		0	–	–	0	–	–	0	–	–
**18–64**	**All-cause mortality**										
		Case–control studies	1	0.46 (0.11–1.89)	NA^1^	1	0.76 (0.07–8.06)	NA^1^	0	–	NA^1^
	**All-cause hospitalization**										
		Case–control studies	2	0.32 (0.19–0.54)	0%	3	0.42 (0.19–0.94)	77%	1	0.73 (0.65–0.83)	NA^1^
	**Influenza/pneumonia hospitalization**										
		Case–control studies	0	–	–	1	0.57 (0.46–0.72)	NA^1^	1	0.88 (0.68–1.14)	NA^1^
	**Influenza-like illness**										
		Cohort studies	1	0.76 (0.50–1.15)	NA^1^	0	–	–	0	–	–
		Case–control studies	0	–	–	1	0.99 (0.97–1.01)	NA^1^	1	1.00 (0.90–1.12)	NA^1^
**≥65**	**All-cause mortality**										
		Cohort studies	3	0.54 (0.37–0.79)	90%	2	0.62 (0.57–0.68)	0%	1	0.70 (0.37–1.31)	NA^1^
		Case–control studies	2	0.39 (0.35–0.43)	0%	2	0.44 (0.36–0.53)	0%	0	–	–
	**All-cause hospitalization**										
		Cohort studies	1	0.83 (0.72–0.95)	NA^1^	0	–	–	1	0.91 (0.71–1.17)	NA^1^
		Case–control studies	2	0.89 (0.81–0.98)	0%	3	0.77 (0.60–0.99)	94%	1	0.66 (0.59–0.74)	NA^1^
	**Influenza/pneumonia hospitalization**										
		Case–control studies	1	0.20 (0.07–0.61)	NA^1^	1	0.55 (0.47–0.66)	NA^1^	1	0.48 (0.32–0.70)	NA^1^
	**Influenza-like illness**										
		Case–control studies	0	–	–	1	0.87 (0.84–0.90)	NA^1^	1	0.82 (0.70–0.96)	NA^1^
**Not reported**	**Influenza/pneumonia hospitalization**										
		Cohort studies	1	1.75 (0.10–32.68)	NA^1^	0	–	–	0	–	–
	**Influenza-like illness**										
		Cohort studies	1	0.34 (0.02–5.85)	NA^1^	0	–	–	0	–	–

OR, Odds ratio; 95% CI, 95% Confidence interval; ^1^Only one study.

### Vaccine effectiveness in elderly aged 65 and older

Crude and adjusted odds ratios for all outcomes in elderly diabetic patients are shown in Table 2. In cohort studies, pooled analysis of adjusted point estimates showed protective effects of influenza vaccination against all-cause mortality (adjusted VE 38%, 95% CI, 32–43%, I^2^ = 0%, n = 2). For the remaining outcomes, only crude point estimates were reported by cohort studies and off-season estimates did not indicate residual confounding here.

Pooled analysis of case–control studies indicated that influenza vaccination prevented all-cause mortality (adjusted VE 56%, 95% CI, 47–64%, I^2^ = 0%, n = 2) and all-cause hospitalization (adjusted VE 23%, 95% CI, 1–40%, I^2^ = 94%, n = 3). Only one study [35] reported data on VE against hospitalization due to influenza or pneumonia (adjusted VE 45%, 95% CI, 34–53%, n = 1), and against ILI (adjusted VE, 13%, 95% CI, 10–16%, n = 1). However, significant VE estimates for all-cause hospitalization, hospitalization due to influenza or pneumonia and for ILI were identified also off-season, often even with higher point estimates than during influenza seasons.

High statistical heterogeneity was observed regarding the outcome all-cause hospitalization among both adults at working age and the elderly. However, we did not find appropriate explanations for these high values, possibly due to the small number of studies.

### Quality of evidence

For working-age adults (18 to 64 years), evidence for a protective effect of influenza vaccination on all-cause mortality was assessed as being of very low quality due to a high imprecision of the estimate. The same quality assessment applied to all-cause hospitalization, with estimates having a high risk of bias and being imprecise. Evidence for effects of influenza vaccination on hospitalization due to influenza or pneumonia and effects on ILI was assessed as being of low quality (Additional file 7 for the GRADE evidence profile).

For elderly patients (65 years and older), evidence on effectiveness for preventing all-cause hospitalization, hospitalization due to pneumonia or influenza, and ILI by influenza vaccination was rated as being of very low quality due to serious risk of bias. For all-cause mortality, quality of evidence was low (Additional file 8 for the GRADE evidence profile).

## Discussion

The evidence on the effectiveness of influenza vaccination in preventing clinical outcomes in patients with diabetes is limited for elderly and adults, and absent for children and adolescents. For elderly patients, the quality of evidence was low for preventing all-cause mortality and very low for all other clinical outcomes. In working-age adults, vaccination was shown effective only against hospitalization due to influenza or pneumonia (quality of evidence: low), whereas no effect was found against any other clinical outcome (quality of evidence: low to very low). In the absence of RCTs, quality of evidence was strongly limited through risk of bias and residual confounding in the observational studies.

### Why the evidence was limited

In our review, we found the currently available evidence on the effectiveness of influenza vaccination in preventing clinical outcomes in diabetic patients to be limited for several reasons. The first important limitation concerns the unavailable data for specific age groups or outcomes. Although a shift towards younger ages has been observed in the manifestation of diabetes in several industrialized countries [40-43], no studies were identified which reported data on influenza VE in diabetic children or adolescents. Additionally, the number of studies on working-age adults and elderly people with diabetes can be regarded as relatively small given the public health relevance of both diabetes and influenza. This ultimately led to wide confidence intervals – at least for some outcomes – which limited the conclusions that could be drawn from these estimates. The lack of studies on safety outcomes related to influenza vaccines when used in diabetic patients was surprising. However, influenza vaccines have been used for decades and safety did not appear to be a serious issue in healthy adults or elderly [30,44]. Regarding diabetic patients, one study compared reactogenicity of trivalent inactivated influenza vaccines with virosomal influenza vaccines in children with diabetes type 1 and found only transient and non-severe adverse events in both groups [45]. Serious adverse events in diabetic patients following influenza vaccination have been reported only in case studies. One study reported two cases of pancreas transplant rejection in patients with diabetes type 1 after immunization against pandemic influenza (H1N1pdm) [46]. Another patient with a family history of type 2 diabetes developed fulminant type 1 diabetes with thrombocytopenia within 7 days after seasonal influenza vaccination [47]. Thus, overall, reactogenicity or severe side effects in patients with diabetes does not seem to be frequent or different from healthy adults.

A second limitation was the choice of case definitions. In the observational studies included in our review, patients with diabetes were mainly identified via disease classification codes. Due to the nature of register-based studies, unspecific outcomes (i.e., outcomes without laboratory confirmation of the diagnosis) were used for identifying influenza disease or related outcomes. Since non-specific outcomes are known to be a poor proxy for influenza, outcomes such as all-cause hospitalization or ILI usually underestimate VE due to dilution effects (since many other causes can lead to the same outcome). In contrast, the outcome ‘influenza/pneumonia hospitalization’ is more specific and might be based on laboratory confirmation by some but not all physicians. However, misclassification is still possible due to selective testing or if the discharge diagnosis was only based on clinical judgment, which can lead to over- or underestimation of VE. Therefore, laboratory confirmation has been advocated as a minimum requirement for studies assessing influenza vaccine efficacy or effectiveness [48-51]. However, such data were not available in the studies analyzed here.

A third limitation concerns the design of available studies. Namely, no RCTs or other experimental studies have been performed so far, to determine the effects of influenza vaccination in the large group of diabetic patients. In observational studies on the effectiveness of influenza vaccination in the presence of comorbidities, two different types of confounding have been described, which affect the comparability of vaccinated and unvaccinated participants. If patients with underlying chronic diseases are more likely to be vaccinated, selection for vaccination is confounded by factors that are also related to clinical outcomes. This scenario is referred to as ‘confounding by indication’ [52]. If no adequate statistical adjustment (e.g., for comorbidities) is made, confounding by indication leads to an underestimation of VE. The alternative scenario is called ‘healthy vaccinee bias’ and refers to a situation when patients who are in better health condition are more likely to be vaccinated [53]. If not corrected for, healthy vaccinee bias leads to an overestimation of VE. We found nearly all of the studies analyzed here to be at risk of confounding by indication. If no correct adjustment had been made for comorbidities, these studies would have underestimated VE. Indication for healthy vaccinee bias was observed in only one study in elderly patients. Since identification of both confounding by indication and healthy vaccine bias is challenging, some authors have suggested using off-season estimates of VE as control period to assess the extent of residual confounding [54]. Any effect of vaccination measured during off-seasons is therefore attributable to confounding. If confounder-adjusted VE estimates do not differ between influenza season and off-season, residual confounding is likely to be present. In our meta-analysis, residual confounding was likely to seriously affect VE estimates in the elderly but only to a small extent in working-age adults. This fits well with the possibility of healthy vaccinee bias in the study by Lau et al. [35], which provided the majority of off-season estimates for this analysis. Adjusting for functional status rather than comorbidities in observational studies might reduce the impact of residual confounding in observational studies [55]; however, ultimately, these problems can only be solved by the conduct of RCTs or by designing other experimental or quasi-experimental studies which minimize bias and confounding.

### Interpretation of meta-analysis results

Our meta-analysis indicated that in working-age adults with diabetes, influenza vaccination prevents hospitalizations but not death or ILI. In contrast to the outcome ‘all-cause hospitalization’, for which a significant off-season effect suggests residual confounding, the more specific outcome ‘influenza/pneumonia hospitalization’ seems more robust and not impaired by residual confounding. For this outcome, only one observational study was identified and showed a VE of 43%, thereby indicating a moderate effectiveness. Why no effect of vaccination was found against all-cause mortality and ILI remains unclear; however, this might be due to the fact that the number of deaths in this age-group is small and that the effect against ILI is diluted through the unspecific clinical case-definition of the outcome itself [56].

Regarding elderly diabetic patients, the results of our meta-analysis showed protection against all-cause mortality and hospitalizations as well as against ILI. However, residual confounding was likely in all outcomes as demonstrated by significant off-season VE estimates except for all-cause mortality. For all-cause mortality, the VE estimate during off-season had a wide 95% CI and the point estimate was not significantly different from the VE point estimate during influenza season. In fact, studies among elderly people have found that VE estimates against all-cause deaths were higher than expected given the observed effectiveness against laboratory-confirmed influenza cases [57,58]. It was concluded that selection bias have led observational studies to greatly exaggerate influenza vaccine benefits in this particular age group [55,59]. Overall, these findings indicate a VE overestimation for all outcomes in elderly diabetic patients, and no precise estimate of a preventive effect can be inferred from these data.

### Strengths and limitations of this review

Our systematic review has several strengths. It is the first systematic evaluation of the literature on this topic, covering published data on more than 170,000 diabetic patients. We performed an outcome- and age group-specific quality assessment of individual studies and assessed the quality of evidence of each reported outcome using the GRADE methodology. We paid particular attention to residual confounding in the observational studies by comparing estimates of VE during influenza seasons to those obtained outside seasons. Furthermore, interpreting and drawing conclusions from the studies was challenging. This was mainly due to the identified limitations of the included studies, namely the lack of RCTs and other experimental studies and the high risk of bias. In addition, only one of the included studies provided data on type of diabetes or glycemic control. Given the wide spectrum of disease severity and treatment success in diabetic patients, influenza VE might differ substantially between patient sub-groups. Factors such as severity of disease and treatment success, differences in study settings and healthcare systems, and methodological variations, might have caused the statistical heterogeneity observed for some outcomes. However, due to the small number of studies it was not possible to investigate this issue by subgroup analysis.

## Conclusions

The WHO and several NITAGs recommend seasonal influenza vaccination of patients with diabetes, regardless of age and severity of the diabetic disease [3-7]. For NITAGs, knowledge about the strength of the vaccine effect and the quality of the underlying evidence are crucial for decision making. However, there are also other relevant key criteria such as disease severity, burden of the disease in a population, and the availability of other preventive measures [60]. Given the large number of people living with diabetes worldwide, it is surprising that it is impossible to determine to which extent diabetic patients benefit from seasonal influenza vaccination.

On the other hand, the absence of alternative effective preventive measures and the good safety profile of seasonal influenza vaccines can still justify the decision to vaccinate the patient even if the quality of evidence on the effectiveness is low. This is particularly true if other underlying risk factors might put the diabetic patient at an increased risk for severe influenza disease.

Given the low to very low quality of evidence related to influenza VE in diabetic adults and elderly and the lack of evidence in children with diabetes, RCTs or carefully conducted quasi-experimental studies using laboratory-confirmed influenza-specific outcomes (e.g., studies using instrumental variable method [61]) are urgently needed to clarify the true effect of influenza vaccination in this important patient population.

## Additional files

Additional file 1:
**Protocol for the systematic review.**
Additional file 2:
**Search strategy.**
Additional file 3:
**Healthy vaccinee bias and confounding by indication in the included studies.**
Additional file 4:
**List of excluded studies.**
Additional file 5:
**Crude, adjusted and off-season point estimates and risk of bias in included studies.**
Additional file 6:
**Application of the Newcastle-Ottawa scale for outcome-specific risk of bias assessment in individual studies.**
Additional file 7:
**GRADE evidence profile in diabetic patients at working age (18–64 years).**
Additional file 8:
**GRADE evidence profile in elderly persons with diabetes (≥65 years).**

## Abbreviations

CI: Confidence interval
GRADE: Grading of recommendations assessment development and evaluation
ICU: Intensive care unit
ILI: Influenza-like illness
NITAG: National immunization technical advisory group
RCT: Randomized controlled trial
VE: Vaccine effectiveness
WHO: World health organization
