# Supplementary material for: Vaccines for the prevention of seasonal influenza in patients with diabetes: systematic review and meta-analysis
Source: BMC Med. 2015 Mar 17;13:53. doi: 10.1186/s12916-015-0295-6 (PMC4373029; doi:10.1186/s12916-015-0295-6)
Supplement: Additional file 1: — Protocol for the systematic review. [file 12916_2015_295_MOESM1_ESM.doc]

**Appendix 1**

**Protocol for systematic review: Vaccines for preventing seasonal influenza in patients with diabetes**

**Reviewer:** Cornelius Remschmidt, Thomas Harder, Ole Wichmann

1. **Review question:** What is the efficacy, effectiveness and safety of seasonal influenza vaccination in patients with diabetes mellitus?
2. **Data sources:**
   1. Electronic data bases: Medline, Embase, Cochrane Central Register of Controlled Trials, ClinicalTrials.gov (from inception to December 31, 2013)
   2. Reference lists of all identified studies
   3. Restrictions: none regarding language or publication type
3. **Search strategy:** The following search strategy will be applied, using the full-text search option via DIMDI:

#1 diabetes

#2 influenza

#3 vaccin*

#4 immuniz*

#5 #3 OR #4

#6 #1 AND #2 AND #5

(restrictions: species: human)

1. **Population/participants:** Study participants of all ages with a diagnosis of diabetes mellitus
2. **Intervention:** Immunization with an approved vaccine against seasonal influenza
3. **Comparator:** Placebo or no vaccination
4. **Study designs:** Studies of any design are eligible
5. **Outcomes:** All clinical outcomes and all types of local and systemic adverse events will be considered as primary outcomes. An influenza infection will be regarded as laboratory-confirmed if data on either PCR or viral culture are reported.
6. **Data extraction:** Two independent reviewers will screen studies by title and abstract for eligibility. Potential disagreements will be solved by discussion or by involving the third reviewer (OW). Identified studies will be retrieved in full text. The following data will be extracted: country, year, study design, age at vaccination, sex, identification of diabetic patients, conflict of interest, vaccine used (brand name, manufacturer), number of vaccinated and unvaccinated participants, proportion of participants lost to follow-up, effect measure (RR, OR, HR) and 95%CI, confounders, confounder-adjusted effect measure, control-period estimate(s). Data extraction forms from the systematic review of influenza vaccine efficacy in HIV-infected patients will be used and adapted according to disease specific variables (e.g. type of diabetes; medical therapy).
7. **Risk of bias assessment:** For randomized controlled trials, risk of bias will be assessed using the Cochrane Risk of Bias Tool. For observational studies (including quasi-experimental studies), the Newcastle-Ottawa Scale will be applied to assess risk of bias.
8. **Data synthesis:** Data will be aggregated in tables, showing study characteristics and effect estimates. Where statistical pooling is appropriate, pooled summary estimates will be calculated. Both fixed-effects and random-effects model will be used. Heterogeneity will be assessed using I² statistic. Separate calculations will be done for each study design.
