# Supplementary material for: Vaccines for the prevention of seasonal influenza in patients with diabetes: systematic review and meta-analysis
Source: BMC Med. 2015 Mar 17;13:53. doi: 10.1186/s12916-015-0295-6 (PMC4373029; doi:10.1186/s12916-015-0295-6)
Supplement: Additional file 2: — Search strategy. [file 12916_2015_295_MOESM2_ESM.doc]

**Appendix 2**

Search strategy for the systematic review on seasonal influenza vaccine effectiveness in patients with diabetes (full text search). According to suggestions of the reviewer, the search strategy defined in the protocol was redone using additional search terms. The following new search strategy was applied:

#1 diabetes

#2 type 1 diabetes

#3 type 2 diabetes

#4 insulin dependent diabetes

#5 gestational diabetes

#6 #1 OR #2 OR #3 OR #4 OR #5

#7 influenza

#8 human influenza

#9 flu

#10 grippe

#11 #7 OR #8 OR #9 OR #10

#12 vaccin*

#13 immuni*

#14 inocula*

#15 #12 OR #13 OR #14

#16 #6 AND #11 AND #15

Databases searched via the German Institute of Medical Documentation and Information surface (text field search; available at: <http://www.dimdi.de/static/en/index.html>): MEDLINE, EMBASE and Cochrane Central Register of Controlled Trials from inception to November 25, 2014; restrictions: species: human.
