# Supplementary material for: Vaccines for the prevention of seasonal influenza in patients with diabetes: systematic review and meta-analysis
Source: BMC Med. 2015 Mar 17;13:53. doi: 10.1186/s12916-015-0295-6 (PMC4373029; doi:10.1186/s12916-015-0295-6)
Supplement: Additional file 3: — Healthy vaccinee bias and confounding by indication in the included studies. [file 12916_2015_295_MOESM3_ESM.doc]

**Appendix 3**

Healthy vaccinee bias and confounding by indication in the included observational studies on seasonal influenza vaccine effectiveness in patients with diabetes

| **Study** | **Healthy vaccinee bias^1^** | **Confounding by indication^2^** |
| --- | --- | --- |
| Colquhoun et al. (1997) | not estimable^3^ | not estimable |
| Gasparini et al. (2013) | unlikely | likely |
| Hak et al. (2002) | unlikely | likely |
| Heymann et al. (2004) | not estimable | not estimable |
| Isotani et al. (2000) | not estimable | not estimable |
| Lau et al. (2013) | likely^4^ | likely^5^ |
| Loojmans et al. (2006) | unlikely | likely |
| Rodriguez et al. (2012) | unlikely | likely |
| Schade et al. (2000) | not estimable | not estimable |
| Selvais et al. (1997) | unlikely | likely |
| Wang et al. (2013) | unlikely | likely |

^1^ indicated by: vaccinated participants were healthier (fewer comorbidities) than unvaccinated participants at study entry (cohort studies) or vaccinated controls were healthier (fewer comorbidities) than unvaccinated controls (case-control studies).

^2^ indicated by: vaccinated participants were sicker (more comorbidities) than unvaccinated participants at study entry (cohort studies) or vaccinated controls were sicker (more comorbidities) than unvaccinated controls (case-control studies).

^3^ not enough data reported on baseline characteristics in vaccinated and unvaccinated participants.

^4^ for elderly (≥ 65 years)

^5^ for working-age adults (18-64 years)
