# Supplementary material for: Vaccines for the prevention of seasonal influenza in patients with diabetes: systematic review and meta-analysis
Source: BMC Med. 2015 Mar 17;13:53. doi: 10.1186/s12916-015-0295-6 (PMC4373029; doi:10.1186/s12916-015-0295-6)
Supplement: Additional file 4: — List of excluded studies. [file 12916_2015_295_MOESM4_ESM.doc]

**Appendix 4**

List of excluded studies on seasonal influenza vaccine effectiveness in patients with diabetes (n=49):

No data on vaccine effectiveness or safety in diabetic patients (n=21):

No new original data (n=8):

Vaccine coverage data only (n=3):

No control group (n=3):

Letter, no new original data (n=2):

Data on diabetic subcohort not extractable (n=2):

Study not found (n=2):

Conference abstract without sufficient data (n=1):

Duplicate (n=7)

**References to studies excluded from this review**

1. Plasai V, Lertmaharit S, Viputsiri OA, Pongpanich S, Panichpathompong U, Tarnmaneewongse V, Baron-Papillon F, Cheunkitmongkol S: **Influenza vaccination among the elderly in Bangkok.** *Southeast Asian J Trop Med Public Health* 2006, **37 Suppl 3:**140-144.

2. Wang CS, Wang ST, Lai CT, Lin LJ, Chou P: **Impact of influenza vaccination on major cause-specific mortality.** *Vaccine* 2007, **25:**1196-1203.

3. Wang CS, Wang ST, Lai CT, Lin LJ, Lee CT, Chou P: **Reducing major cause-specific hospitalization rates and shortening hospital stays after influenza vaccination.** *Clin Infect Dis* 2004, **39:**1604-1610.

4. Davies P, Nwokoro C, Leigh M: **Vaccinations against influenza and pneumococcus in children with diabetes: telephone questionnaire survey.** *BMJ* 2004, **328:**203.

5. Sanchez Munoz-Torrero JF, Saponi Cortes JM, Ortiz Descante C, Ojeda Garcia Escribano I, Sanchez Sanchez T, Perez Reyes F, Martin Ruiz C, Costo Campoamor A: **[Utilization and effectiveness of the flu vaccination in the prevention of the hospitalization induced by cardiorespiratory decompensation in high-risk patients in Caceres].** *Rev Clin Esp* 2003, **203:**363-367.

6. Smith SA, Poland GA, American Diabetes A: **Immunization and the prevention of influenza and pneumococcal disease in people with diabetes.** *Diabetes Care* 2003, **26 Suppl 1:**S126-128.

7. Valdez R, Narayan KM, Geiss LS, Engelgau MM: **Impact of diabetes mellitus on mortality associated with pneumonia and influenza among non-Hispanic black and white US adults.** *Am J Public Health* 1999, **89:**1715-1721.

8. Nichol KL, Wuorenma J, von Sternberg T: **Benefits of influenza vaccination for low-, intermediate-, and high-risk senior citizens.** *Arch Intern Med* 1998, **158:**1769-1776.

9. el-Madhun AS, Cox RJ, Seime A, Sovik O, Haaheim LR: **Systemic and local immune responses after parenteral influenza vaccination in juvenile diabetic patients and healthy controls: results from a pilot study.** *Vaccine* 1998, **16:**156-160.

10. Ho TY, Huang KY, Huang TT, Huang YS, Ho HC, Chou P, Lin CH, Wei CK, Lian WC, Chen TC, et al: **The impact of influenza vaccinations on the adverse effects and hospitalization rate in the elderly: a national based study in an Asian country.** *PLoS One* 2012, **7:**e50337.

11. Vila-Corcoles A, Rodriguez T, de Diego C, Ochoa O, Valdivieso A, Salsench E, Ansa X, Badia W, Saun N, Group ES: **Effect of influenza vaccine status on winter mortality in Spanish community-dwelling elderly people during 2002-2005 influenza periods.** *Vaccine* 2007, **25:**6699-6707.

12. Watkins J: **Effectiveness of influenza vaccination policy at targeting patients at high risk of complications during winter 1994-5: cross sectional survey.** *BMJ* 1997, **315:**1069-1070.

13. Wiwanitkit V: **Influenza vaccination for diabetic case.** *Diabetes & Metabolic Syndrome: Clinical Research & Reviews* 2010, **4:**60-61.

14. Nowakowska M, Buczkowska E: **Infectious diseases and vaccination in adolescent diabetic patients [Schorzenia infekcyjne i szczepienia ochronne u mlodocianych pacjentów chorych na cukrzyce].** *Family Medicine and Primary Care Review* 2007, **9:**266-273.

15. Satman I, Akalin S, Cakir B, Altinel S, dia VAXSG: **The effect of physicians' awareness on influenza and pneumococcal vaccination rates and correlates of vaccination in patients with diabetes in Turkey: an epidemiological Study "diaVAX".** *Hum Vaccin Immunother* 2013, **9:**2618-2626.

16. Saah AJ, Neufeld R, Rodstein M, La Montagne JR, Blackwelder WC, Gross P, Quinnan G, Kaslow RA: **Influenza vaccine and pneumonia mortality in a nursing home population.** *Arch Intern Med* 1986, **146:**2353-2357.

17. Abdallah J, Anna K, Hassan T, Jain A: **Vaccination Outcomes in Inflammatory Bowel Disease. Gastroenterology, 2014. 146(5): p. 170.** 2014.

18. Ergonul O, Alan S, Ak O, Sargin F, Kanturk A, Gunduz A, Engin D, Oncul O, Balkan, II, Ceylan B, et al: **Predictors of fatality in pandemic influenza A (H1N1) virus infection among adults.** *BMC Infect Dis* 2014, **14:**317.

19. Gomez-Gomez A, Magana-Aquino M, Bernal-Silva S, Araujo-Melendez J, Comas-Garcia A, Alonso-Zuniga E, Torres-Torres E, Noyola DE: **Risk factors for severe influenza A-related pneumonia in adult cohort, Mexico, 2013-14.** *Emerg Infect Dis* 2014, **20:**1554-1558.

20. Lau D, Eurich DT, Majumdar SR, Katz A, Johnson JA: **Working-age adults with diabetes experience greater susceptibility to seasonal influenza: a population-based cohort study.** *Diabetologia* 2014, **57:**690-698.

21. McDonald HI, Nitsch D, Millett ER, Sinclair A, Thomas SL: **New estimates of the burden of acute community-acquired infections among older people with diabetes mellitus: a retrospective cohort study using linked electronic health records.** *Diabet Med* 2014, **31:**606-614.

22. Michiels B, Govaerts F, Remmen R, Vermeire E, Coenen S: **A systematic review of the evidence on the effectiveness and risks of inactivated influenza vaccines in different target groups.** *Vaccine* 2011, **29:**9159-9170.

23. Salemi S, D'Amelio R: **Are anti-infectious vaccinations safe and effective in patients with autoimmunity?** *Int Rev Immunol* 2010, **29:**270-314.

24. Wiwanitkit V: **Usefulness of influenza vaccination in different groups of diabetic patients.** *Diabetes and Metabolic Syndrome: Clinical Research and Reviews* 2011, **5:**216-217.

25. Zuccotti G: **Safety of a virosomal adjuvanted influenza vaccine in children suffering from chronic disease.** *Open Vaccine Journal* 2010, **3:**108-113.

26. Lösch H: **PRISMA study proves: Flu vaccination reduces mortality and complications in diabetics by more than 50 percent.** *Krankenhaushygiene und Infektionsverhutung* 2009, **31:**25.

27. Sadler M, Heyworth N: **Influenza vaccination in diabetes: Is it evidence based?** *Practical Diabetes International* 1998, **15:**193-194.

28. American Association of Diabetes E: **Vaccination practices for hepatitis B, influenza, and pneumococcal disease for people with diabetes.** *Diabetes Educ* 2014, **40:**122-124.

29. Fedson DS: **Influenza vaccination effectiveness, unmeasured confounding, and immunomodulatory treatment.** *J Infect Dis* 2014, **209:**1300-1301.

30. Lu PJ, Gonzalez-Feliciano A, Ding H, Bryan LN, Yankey D, Monsell EA, Greby SM, Euler GL: **Influenza A (H1N1) 2009 monovalent and seasonal influenza vaccination among adults 25 to 64 years of age with high-risk conditions--United States, 2010.** *Am J Infect Control* 2013, **41:**702-709.

31. Jimenez-Garcia R, Mayo-Montero E, Hernandez-Barrera V, Garrido PC, Martinez-Hernandez D, de Miguel AG: **Influenza vaccination among diabetic adults: related factors and trend from 1993 to 2001 in Spain.** *Diabetes Care* 2005, **28:**2031-2033.

32. Roubini A, Jego M, Larger E: **[Vaccination against influenza in diabetics: survey at the end of winter 1996-97].** *Presse Med* 1997, **26:**1970.

33. Zuccotti GV, Scaramuzza A, Riboni S, Mameli C, Pariani E, Tanzi E, Zanetti A, Radaelli G: **Long-lasting immunogenicity of a virosomal vaccine in older children and young adults with type I diabetes mellitus.** *Vaccine* 2009, **27:**5357-5362.

34. Jackson LA, Holmes SJ, Mendelman PM, Huggins L, Cho I, Rhorer J: **Safety of a trivalent live attenuated intranasal influenza vaccine, FluMist, administered in addition to parenteral trivalent inactivated influenza vaccine to seniors with chronic medical conditions.** *Vaccine* 1999, **17:**1905-1909.

35. Dorrell L, Hassan I, Marshall S, Chakraverty P, Ong E: **Clinical and serological responses to an inactivated influenza vaccine in adults with HIV infection, diabetes, obstructive airways disease, elderly adults and healthy volunteers.** *Int J STD AIDS* 1997, **8:**776-779.

36. Vinicor F: **Flu vaccination decreases relative morbidity risk for people with diabetes.** *J Med Assoc Ga* 1998, **87:**330.

37. Ong R, Kwa AL, Lee W: **Dual pneumococcal and influenza vaccination in elderly patients with chronic illnesses: protective benefits overestimated?** *Clin Infect Dis* 2011, **52:**558-559; author reply 559.

38. Groenwold RH, Hoes AW, Hak E: **Impact of influenza vaccination on mortality risk among the elderly.** *Eur Respir J* 2009, **34:**56-62.

39. McLean HQ, Meece JK, Belongia EA: **Influenza vaccination and risk of hospitalization among adults with laboratory confirmed influenza illness.** *Vaccine* 2014, **32:**453-457.

40. No_author: **Preventing pneumococcal infections and influenza in diabetic patients.** *Journal of Respiratory Diseases* 2000, **21:**708-711.

41. Koutsovasilis AG: **Prevalence and impact of influenza and pneumococcal vaccination in type 2 diabetes mellitus patients. Diabetes, 2014. 63: p. 642.** 2014.

42. Vamos EP, Pape UJ, Curcin V, Harris MJ, Valabhji J, Majeed A, Millett C: **Influenza vaccine effectiveness against hospitalisation and death in people with Type 2 diabetes.** pp. 74-75: WILEY-BLACKWELL; 2014:74-75.
