# Supplementary material for: Vaccines for the prevention of seasonal influenza in patients with diabetes: systematic review and meta-analysis
Source: BMC Med. 2015 Mar 17;13:53. doi: 10.1186/s12916-015-0295-6 (PMC4373029; doi:10.1186/s12916-015-0295-6)
Supplement: Additional file 5: — Crude, adjusted and off-season point estimates and risk of bias in included studies. [file 12916_2015_295_MOESM5_ESM.doc]

**Appendix 5**

Crude, adjusted and off-season point estimates and risk of bias in included observational studies. Point estimates are reported as odd ratio (OR) with 95% confidence interval (CI). Risk of bias for specific outcomes is expressed as considered judgment.

|  |  |  |  |  |  |  |  |
| --- | --- | --- | --- | --- | --- | --- | --- |
| **Age-group (years)** |  | **Outcome/**  **study design** | **Author** | **Crude OR (95% CI)** | **Adjusted OR  (95% CI)** | **Control period: Off-season adjusted OR (95% CI)** | **Risk of  bias** |
| **0-17** | **No studies** | | - | - | - | - | - |
| **18-64** | **All-cause mortality** | | |  |  |  |  |
|  |  | Case-control studies | Looijmans | 0.46 (0.11-1.89) | 0.76 (0.07-8.06)^1^ | - | Low |
|  | **Hospitalization OR death** | | |  |  |  |  |
|  |  | Case-control studies | Looijmans | 0.37 (0.21-0.64) | 0.28 (0.15-0.54)^1^ | - | Low |
|  | **All-cause hospitalization** | | |  |  |  |  |
|  |  | Case-control studies | Colquhoun^2^ | 0.19 (0.05-0.70) | 0.21 (0.05-0.81)^3^ | - | High |
|  |  |  | Looijmans | 0.35 (0.20-0.64) | 0.30 (0.15-0.61)^1^ | - | Low |
|  |  |  | Lau | - | 0.72 (0.68-0.76)^4^ | 0.73 (0.65-0.83)^4,5^ | High |
|  | **Influenza/pneumonia hospitalization** | | |  |  |  |  |
|  |  | Case-control studies | Lau | - | 0.57 (0.46-0.72)^4^ | 0.88 (0.68-1.14)^4,5^ | Low |
|  | **Influenza-like illness** | | |  |  |  |  |
|  |  | Cohort studies | Selvais^6^ | 0.76 (0.50-1.15)^7^ | - | - | High |
|  |  | Case-control studies | Lau | - | 0.99 (0.97-1.01)^4^ | 1.00 (0.90-1.12)^4,5^ | Low |
| **≥ 65** | **All-cause mortality** | | |  |  |  |  |
|  |  | Cohort studies | Heymann^8^ | 0.34 (0.26-0.45) | - | - | High |
|  |  |  | Rodriguez-Blanco | 0.83 (0.60-1.14) | 0.67 (0.47-0.96)^9^ | 0.70 (0.37-1.31)^10^ | Low |
|  |  |  | Schade^11^ | 0.57 (0.54-0.60) | 0.62 (0.57-0.67)^12^ | - | High |
|  |  | Case-control studies | Looijmans | 0.36 (0.19-0.68) | 0.44 (0.20-0.96)^1^ | - | Low |
|  |  |  | Wang | 0.39 (0.35-0.45) | 0.44 (0.36-0.54)^13^ | - | Low |
|  | **Hospitalization OR death** | | |  |  |  |  |
|  |  | Cohort studies | Hak | 0.64 (0.49-0.84) | 0.63 (0.40-0.99)^14^ | - | Low |
|  |  | Case-control studies | Looijmans | 0.60 (0.37-0.98) | 0.61 (0.35-1.05)^1^ | - | Low |
|  | **All-cause hospitalization** | | |  |  |  |  |
|  |  | Cohort studies | Heymann^8^ | 0.83 (0.72-0.95) | - | 0.91 (0.71-1.17)^15^ | High |
|  |  | Case-control studies | Looijmans | 1.0 (0.49-2.07) | 0.86 (0.40-1.88)^1^ | - | Low |
|  |  |  | Lau | - | 0.67 (0.64-0.70)^4^ | 0.66 (0.59-0.74)^4,5^ | High |
|  |  |  | Wang | 0.89 (0.81-0.98) | 0.88 (0.81-0.96)^12^ | - | Low |
|  | **Influenza/pneumonia hospitalization** | | |  |  |  |  |
|  |  | Case-control studies | Gasparini | 0.20 (0.07-0.62)^7^ | - | - | High |
|  |  |  | Lau | - | 0.55 (0.47-0.66)^4^ | 0.48 (0.32-0.70)^4,5^ | High |
|  | **Influenza-like illness** | | |  |  |  |  |
|  |  | Case-control studies | Lau | - | 0.87 (0.84-0.90)^4^ | 0.82 (0.70-0.96)^4,5^ | High |
| **not reported** | **Influenza/pneumonia hospitalization** | | |  |  |  |  |
|  |  | Cohort studies | Isotani | 1.77 (0.10-32.68)^7^ | - | - | High |
|  | **Influenza-like illness** | | |  |  |  |  |
|  |  | Cohort studies | Isotani | 0.34 (0.02-5.85)^7^ | - | - | High |
| OR, odds ratio; 95% CI, 95% confidence interval ^1^ Adjusted for age, sex , National Health Insurance, mean GP visits, mean prescriptions, specialists care, hospitalization, comorbidities ^2^ Age ranged from ≤19 to 85 years; however, since majority of cases and controls were < 65 years this study was allocated to studies on working-age adults ^3^ Adjusted for age, sex, year, type of diabetes, comorbidities, no of GP visits and no of hospital admissions  ^4^  Adjusted for sex, age, income, pneumococcal vaccine receipt, number of medical visits (previous year), number of ADGs as well as matching for age, sex, residence ^5^ Point estimates before and after influenza season were pooled first ^6^ mean age was 56.3 years (standard deviation (SD) ± 15.9), therefore this study was allocated to working-age adults ^7^ OR calculated from original data ^8^ Point estimates for women and men were pooled ^9^ Adjusted for age, sex , chronic heart disease (CHD), immunosuppression, number of outpatient visits, interaction (CHD x immunosuppression) and propensity score quintiles ^10^ Adjusted for age, sex , hypertension, immunosuppression, smoking and propensity score quintiles ^11^ Point estimates from two seasons were pooled ^12^ Adjusted for age, sex, number of inpatient diabetes diagnoses per year, comorbidities (ischemic disease diagnoses, COPD, chronic renal failure diagnoses) ^13^ Adjusted hazard ratios, adjusted for age, sex and comorbidities ^14^ Adjusted for age, sex, comorbidities and baseline health care use ^15^ Crude estimates, stratified for age-groups and sex | | | | | | | |
