# Supplementary material for: Vaccines for the prevention of seasonal influenza in patients with diabetes: systematic review and meta-analysis
Source: BMC Med. 2015 Mar 17;13:53. doi: 10.1186/s12916-015-0295-6 (PMC4373029; doi:10.1186/s12916-015-0295-6)
Supplement: Additional file 6: — Application of the Newcastle-Ottawa scale for outcome-specific risk of bias assessment in individual studies. [file 12916_2015_295_MOESM6_ESM.doc]

**Appendix 6**

Application of the Newcastle-Ottawa scale for outcome-specific and age-group specific risk of bias assessment in individual studies
