# Supplementary material for: Vaccines for the prevention of seasonal influenza in patients with diabetes: systematic review and meta-analysis
Source: BMC Med. 2015 Mar 17;13:53. doi: 10.1186/s12916-015-0295-6 (PMC4373029; doi:10.1186/s12916-015-0295-6)
Supplement: Additional file 7: — GRADE evidence profile in diabetic patients at working age (18–64 years). [file 12916_2015_295_MOESM7_ESM.doc]

**Appendix 7**

GRADE evidence profile for efficacy, effectiveness and safety of influenza vaccination in diabetic patients at working age (18-64 years)

| **Quality assessment** | | | | | | | **No of patients** | | **Effect** | | **Quality** | **Importance** |
| --- | --- | --- | --- | --- | --- | --- | --- | --- | --- | --- | --- | --- |
|  |  |  |  |  |  |  |  |  |  |  |  |  |
| **No of studies** | **Design** | **Risk of bias** | **Inconsistency** | **Indirectness** | **Imprecision** | **Other considerations** | **Vaccination against influenza** | **Control** | **Relative (95% CI)** | **Absolute** |  |  |
| **All-cause mortality** | | | | | | | | | | | | |
| 1 | observational studies^1^ | no serious risk of bias | no serious inconsistency | no serious indirectness | very serious^2^ | none | - | | OR 0.76 (0.07 to 8.06)^3^ | - | ⊕OOO VERY LOW | CRITICAL |
|  |  |  |  |  |  |  |  | 0.05% |  | 0 fewer per 1000 (from 0 fewer to 4 more) |  |  |
|  |  |  |  |  |  |  |  | 0.5% |  | 1 fewer per 1000 (from 5 fewer to 34 more) |  |  |
|  |  |  |  |  |  |  |  | 1% |  | 2 fewer per 1000 (from 9 fewer to 65 more) |  |  |
| **All-cause hospitalisation** | | | | | | | | | | | | |
| 3 | observational studies^1^ | serious^4^ | serious^5^ | no serious indirectness | serious^6^ | none | - | | OR 0.42 (0.19 to 0.94)^7^ | - | ⊕OOO VERY LOW | CRITICAL |
|  |  |  |  |  |  |  |  | 1% |  | 6 fewer per 1000 (from 1 fewer to 8 fewer) |  |  |
|  |  |  |  |  |  |  |  | 5% |  | 28 fewer per 1000 (from 3 fewer to 40 fewer) |  |  |
|  |  |  |  |  |  |  |  | 10% |  | 55 fewer per 1000 (from 5 fewer to 79 fewer) |  |  |
| **Influenza/pneumonia hospitalization** | | | | | | | | | | | | |
| 1 | observational studies^1^ | no serious risk of bias | no serious inconsistency | no serious indirectness | no serious imprecision | none | - | | OR 0.57 (0.46 to 0.72)^8^ | - | ⊕⊕OO LOW | CRITICAL |
|  |  |  |  |  |  |  |  | 1% |  | 4 fewer per 1000 (from 3 fewer to 5 fewer) |  |  |
|  |  |  |  |  |  |  |  | 5% |  | 21 fewer per 1000 (from 13 fewer to 26 fewer) |  |  |
|  |  |  |  |  |  |  |  | 10% |  | 40 fewer per 1000 (from 26 fewer to 51 fewer) |  |  |
| **Influenza-like illness** | | | | | | | | | | | | |
| 1 | observational studies^1^ | no serious risk of bias | no serious inconsistency | no serious indirectness | no serious imprecision | none | - | | OR 0.99 (0.97 to 1.01)^8^ | - | ⊕⊕OO LOW | CRITICAL |
|  |  |  |  |  |  |  |  | 1% |  | 0 fewer per 1000 (from 0 fewer to 0 more) |  |  |
|  |  |  |  |  |  |  |  | 5% |  | 0 fewer per 1000 (from 1 fewer to 0 more) |  |  |
|  |  |  |  |  |  |  |  | 10% |  | 1 fewer per 1000 (from 3 fewer to 1 more) |  |  |

^1^ case-control
^2^ Very wide confidence interval including large benefit and severe harm.
^3^ OR adjusted for age, sex , NH-Insurance, mean GP visits, mean prescriptions, specialists care, hospitalisation, comorbidities.
^4^ 2 out of 3 studies were at high risk of bias.
^5^ Adjusted odds ratios ranged from 0,21 to 0.72.
^6^ Wide confidence interval around the pooled estimate
^7^ OR adjusted in all three studies at least for age, sex and comorbidities.
^8^ OR adjusted for sex, age (20-year age bands, income, pneumoccal vaccine receipt, n medical visits (previous year), number of ADGs (minor+major), month, year + matching for age, sex, residence.
