# Supplementary material for: Vaccines for the prevention of seasonal influenza in patients with diabetes: systematic review and meta-analysis
Source: BMC Med. 2015 Mar 17;13:53. doi: 10.1186/s12916-015-0295-6 (PMC4373029; doi:10.1186/s12916-015-0295-6)
Supplement: Additional file 8: — GRADE evidence profile in elderly persons with diabetes (≥65 years). [file 12916_2015_295_MOESM8_ESM.doc]

**Appendix 8**

GRADE evidence profile for efficacy, effectiveness and safety of influenza vaccination in elderly persons with diabetes (≥ 65 years)

| **Quality assessment** | | | | | | | **No of patients** | | **Effect** | | **Quality** | **Importance** |
| --- | --- | --- | --- | --- | --- | --- | --- | --- | --- | --- | --- | --- |
|  |  |  |  |  |  |  |  |  |  |  |  |  |
| **No of studies** | **Design** | **Risk of bias** | **Inconsistency** | **Indirectness** | **Imprecision** | **Other considerations** | **Vaccination against influenza** | **Control** | **Relative**  **(95% CI)** | **Absolute** |  |  |
| **All-cause mortality** | | | | | | | | | | | | |
| 2 | observational studies^1^ | no serious risk of bias | no serious inconsistency | no serious indirectness | no serious imprecision | none | - | | OR 0.44 (0.36 to 0.53)^2^ | - | ⊕⊕OO LOW | CRITICAL |
|  |  |  |  |  |  |  |  | 0.5% |  | 3 fewer per 1000 (from 2 fewer to 3 fewer) |  |  |
|  |  |  |  |  |  |  |  | 1% |  | 6 fewer per 1000 (from 5 fewer to 6 fewer) |  |  |
|  |  |  |  |  |  |  |  | 2% |  | 11 fewer per 1000 (from 9 fewer to 13 fewer) |  |  |
| **All-cause hospitalisation** | | | | | | | | | | | | |
| 3 | observational studies^1^ | serious^3^ | no serious inconsistency | no serious indirectness | no serious imprecision | none | - | | OR 0.77 (0.6 to 0.99)^4^ | - | ⊕OOO VERY LOW | CRITICAL |
|  |  |  |  |  |  |  |  | 2% |  | 5 fewer per 1000 (from 0 fewer to 8 fewer) |  |  |
|  |  |  |  |  |  |  |  | 10% |  | 21 fewer per 1000 (from 1 fewer to 37 fewer) |  |  |
|  |  |  |  |  |  |  |  | 20% |  | 39 fewer per 1000 (from 2 fewer to 70 fewer) |  |  |
| **Influenza/pneumonia hospitalization** | | | | | | | | | | | | |
| 1 | observational studies^1^ | serious^3^ | no serious inconsistency | no serious indirectness | no serious imprecision | none | - | | OR 0.55 (0.47 to 0.66)^5^ | - | ⊕OOO VERY LOW | CRITICAL |
|  |  |  |  |  |  |  |  | 2% |  | 9 fewer per 1000 (from 7 fewer to 10 fewer) |  |  |
|  |  |  |  |  |  |  |  | 10% |  | 42 fewer per 1000 (from 32 fewer to 50 fewer) |  |  |
|  |  |  |  |  |  |  |  | 20% |  | 79 fewer per 1000 (from 58 fewer to 95 fewer) |  |  |
| **Influenza-like illness** | | | | | | | | | | | | |
| 1 | observational studies^1^ | serious^3^ | no serious inconsistency | no serious indirectness | no serious imprecision | none | - | | OR 0.87 (0.84 to 0.9)^5^ | - | ⊕OOO VERY LOW | CRITICAL |
|  |  |  |  |  |  |  |  | 2% |  | 3 fewer per 1000 (from 2 fewer to 3 fewer) |  |  |
|  |  |  |  |  |  |  |  | 10% |  | 12 fewer per 1000 (from 9 fewer to 15 fewer) |  |  |
|  |  |  |  |  |  |  |  | 20% |  | 21 fewer per 1000 (from 16 fewer to 26 fewer) |  |  |

^1^ case-control
^2^ OR in the 2 studies adjusted at least for age, sex and comorbidities.
^3^ Although estimates were adjusted for confounders, data from control periods (off-season) suggest the presence of residual confounding.
^4^ OR adjusted in all three studies at least for age, sex and comorbidities.
^5^ OR adjusted for sex, age (20-year age bands, income, pneumoccal vaccine receipt, n medical visits (previous year), number of ADGs (minor+major), month, year + matching for age, sex, residence.
